# Which B2 Fractures Can Be Treated with ORIF? Validation of the “Beyond the Vancouver Classification”

**DOI:** 10.3390/medicina61071138

**Published:** 2025-06-24

**Authors:** Karl Stoffel, Martin Clauss, Marlene Mauch

**Affiliations:** 1 Department of Orthopaedics and Traumatology, University Hospital Basel, 4031 Basel, Switzerland; karl.stoffel@usb.ch (K.S.); martin.clauss@usb.ch (M.C.); 2 Center for Musculoskeletal Infections (ZMSI), University Hospital Basel, 4031 Basel, Switzerland; 3 Department of Biomedical Engineering, University of Basel, 4123 Allschwil, Switzerland

**Keywords:** Vancouver classification, periprosthetic fractures, loose stem, arthroplasty, ORIF, femoral fractures, hip fractures, fracture fixation

## Abstract

*Background and objectives:* The objective was to validate the Beyond the Vancouver classification. Based on this algorithm, it was hypothesized that cemented polished tapered stems with an intact cement mantle and cementless stable stems with defined criteria could be classified as stable and therefore treated with open reduction and internal fixation (ORIF). *Materials and Methods:* This retrospective, single-center cohort study re-analyzed patients initially diagnosed with Vancouver type B2 fractures treated with ORIF between 2007 and 2020. Clinical and radiological outcomes were extracted from medical reports. A combined radiological and clinical score was used as the main outcome measure. Patients categorized according to the Beyond the Vancouver classification were compared for functional outcome. *Results:* 42 patients (25 male, 17 female) with a median (range) age of 83 years (75–88 years) and follow-up time of 25 weeks (12–35 weeks) were reviewed. It was found that ORIF achieved excellent or good results in 81% of cases for stems classified as stable (n = 16) and in 30% of cases for stems classified as loose (n = 23). Successful cases (30%), although classified as loose, all had the same fracture pattern: an intact greater trochanter and a fracture fragment attached laterally to the stem with distal fixation of the stem. *Conclusions:* This case series suggests that certain Vancouver B2 fractures can be treated with ORIF. The Beyond the Vancouver classification may support the categorization of ‘stable’ and ‘loose’ stems. The validity of the algorithm was supported by the observation that ORIF provided excellent and good results for the majority of stems classified as ‘stable’, but poor results for stems classified as ‘loose’. Furthermore, the fracture pattern has been shown to be a crucial factor that should be considered when treating distally fixed cementless stems. The classification was therefore expanded to include the specific fracture patterns in cementless distally fixed stems that can be successfully treated with ORIF. The Beyond the Vancouver classification can provide further guidance in the identification of ‘loose’ or ‘stable’ stems.

## 1. Introduction

The incidence of femoral periprosthetic fractures (PPF) after total hip replacement (THR) is up to 1.4 per 1000 primary THRs [1], with high morbidity and mortality rates [2,3].

The correct treatment is of paramount importance in the reduction in postoperative complications and the enhancement of outcomes. The success of the treatment is contingent upon the quality of the pre-operative planning and the surgeon’s judicious decision during surgery. A number of treatment algorithms have been documented in the relevant literature, with the aim of defining the correct treatment strategy [4,5,6,7,8]. It is evident that the Vancouver classification [9] is currently one of the most widely utilized and endorsed classification systems for PPFs. Subsequent to this, the classification system has been expanded into the Unified classification system [5]. This system classifies fractures into five subtypes according to the fracture location, implant stability and residual bone stock. Fractures of type A are located in the trochanteric region; fractures of type B are located around the stem; and fractures of type C are located below the tip of the stem Vancouver Type B fractures are the most prevalent around a femoral prosthetic stem [10]. Type B fractures are categorized further according to implant stability and bone quality. This categorization system includes the following types: B1 (well-fixed stem), B2 (loose stem, good proximal bone quality), and B3 (poor bone quality or severely comminated proximal bone) [11].

It is widely accepted that Vancouver B1 fractures can be treated with open reduction and internal fixation (ORIF), with cerclage alone or cerclage and plate fixation [12,13]. Whilst contemporary guidelines advocate for the utilization of long-stemmed prostheses in all Vancouver B2 and B3 PFF cases [14], recent research papers propose that ORIF could yield a comparable outcome in specific B2 fractures [12,15,16,17]. It is widely accepted that open reduction internal fixation (ORIF) carries a reduced risk profile, characterized by reduced surgery times, minimized blood loss, and a less complex procedure [18,19]. Consequently, it is recommended as the preferred therapeutic option.

Although the differentiation between B1 and B2 fractures appears straightforward, fractures with a loose prosthetic stem can be challenging to classify in certain instances and are easily missed [10,11,20]. It is reported by several authors that the use of ORIF has been unsuccessful, as loose stems are considered stable, resulting in an unsatisfactory treatment outcome [7,8]. Consequently, precise classification and the identification of loose stems are paramount for determining the most suitable therapeutic approach. The implementation of more precise clinical and radiological criteria has been requested in order to facilitate classification [14]. In response to the aforementioned issue, Stoffel et al. (2020) [11] developed an algorithmic approach to more accurately identify loose stems in PPFs. This approach, the Beyond the Vancouver classification, has been shown to enhance the accuracy of stem identification in PPFs. It extends the existing Vancouver classification of type B fractures with the patient’s clinical history (pain), implant design (cemented, non-cemented), comprehensive radiographic analysis (subsidence, osteolysis, fracture details), and intraoperative findings [11].

The objective of the present study was to validate this Beyond the Vancouver classification [11]. It was hypothesized that, based on the algorithm, fractures initially classified as B2 (loose) could be classified as stable (B2) and treated with ORIF if they were cemented, polished, tapered stems with an intact cement mantle allowing anatomical reduction, or cementless stems with specific fracture patterns.

## 2. Materials and Methods

### 2.1. Study Design

**Figure 1:**
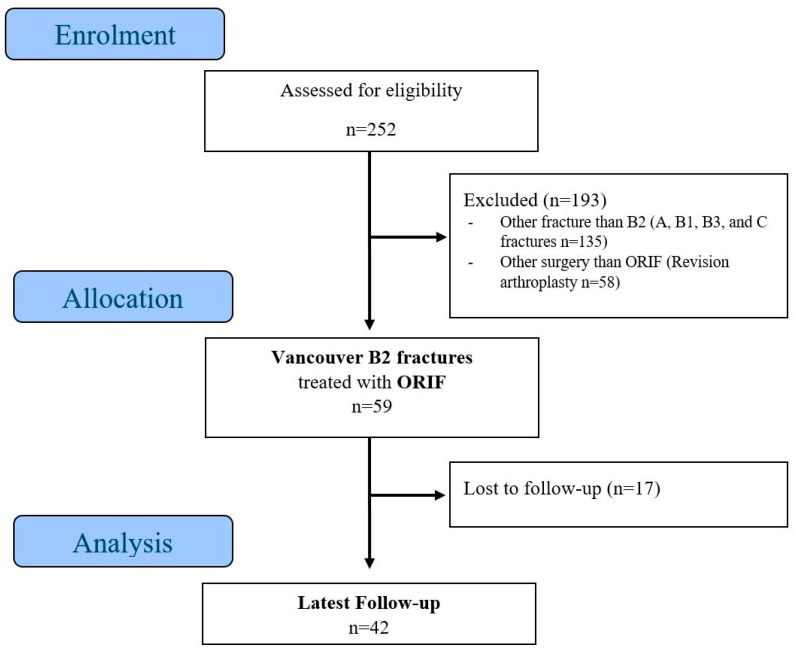
Flow chart of study population.

In this retrospective, single-center study 252, patients who underwent revision arthroplasty (RA) or open reduction internal fixation (ORIF) for the treatment of periprosthetic fractures (PPF) between 2007 and 2020 at the University Hospital of Basel were identified and extracted from the internal clinical information system (CIS). Excluded from the analyses were patients with Vancouver fractures classified other than B2, pathological fractures as result of tumor or metastasis, other treatment than ORIF (revision arthroplasty), or a documented consent dissent for use of clinical data for research. It was imperative that the follow-up examination be conducted a minimum of three months subsequent to the ORIF procedure. The three-month follow-up examination is regarded as an appropriate timeframe for the evaluation of the healing process and the fixation of the implant [21]. Finally, 42 patients whose fractures were classified as Vancouver type B2 and treated with ORIF were the subject of further analysis. The demographic composition of the study population is illustrated in Figure 1. The study was approved by the local ethics committee (EKNZ-2023-00029).

The demographic details of the final 42 subjects included in the study are delineated in Table 1.

### 2.2. Radiographic and Clinical Evaluation

Radiological and clinical variables were retrospectively extracted from the medical records. The radiological assessment was conducted using anteroposterior and axial radiographs of the proximal femur. Two independent surgeons reviewed the preoperative (fracture), perioperative, immediate postoperative and follow-up X-rays to ascertain the intricacies of the arthroplasty, assess implant stability, and ascertain fracture classification in accordance with the Beyond Vancouver Classification framework [11]. In instances of discrepancy between the classifications, consultation with an additional reviewer was initiated. Bone loss due to osteolysis and subsidence was examined in order to assess stem stability. Stem subsidence was measured at each time point, with the distance between the tip of the greater trochanter and the shoulder of the stem being recorded. A measurement of 5 mm or more was considered to be clinically significant and beyond the measurement error [22]. Healing was defined as a radiological union [16]. An evaluation was conducted to ascertain the presence of any signs indicative of a broken or deficient cement mantle, with particular reference to its effect on the cement-bone interface. This evaluation was conducted in accordance with the qualitative approach previously outlined by Stoffel et al. [11].

A further review of the medical records was conducted for patients, with particular attention given to the following: baseline demographics; reported intraoperative loose stems; the incidence of complications; pain; and mobility. The measurement of these parameters included the use of walking aids and walking distance.

### 2.3. Primary and Secondary Outcome

In accordance with the radiological assessment and clinical information, the functional outcome score was used as the primary outcome measure. The criteria were adapted and extended from those proposed by Beals and Tower (1996) [23]. Further details are provided in Table 2: an excellent result was achieved when the endoprosthesis was stable (no osteolysis, subsidence < 5 mm), the fracture has healed, the patient could walk without or with minimal restriction and without limping, and had minimal or no pain. The secondary outcome measure was the incidence of complications.

### 2.4. Statistical Analysis

The subsequent analysis of the data was conducted using descriptive statistics. A comparison was made between the categorized subgroups (stable, loose) as defined by the Beyond the Vancouver Classification and the functional outcome score (adapted from Beals and Tower, 1996) [23]. This was achieved by means of a Mann–Whitney U test. In order to assess the relevance of the result, effect sizes were calculated and interpreted according to Cohen’s classification, which categorizes effect sizes as follows: small (d < 0.3), intermediate (d = 0.3–0.5), and large (d > 0.5) [24]. *p*-values below 0.05 were considered to indicate significant differences. All analyses were performed using SPSS Version 29 (IBM Corporation, Armonk, NY, USA).

## 3. Results

**Figure 2:**
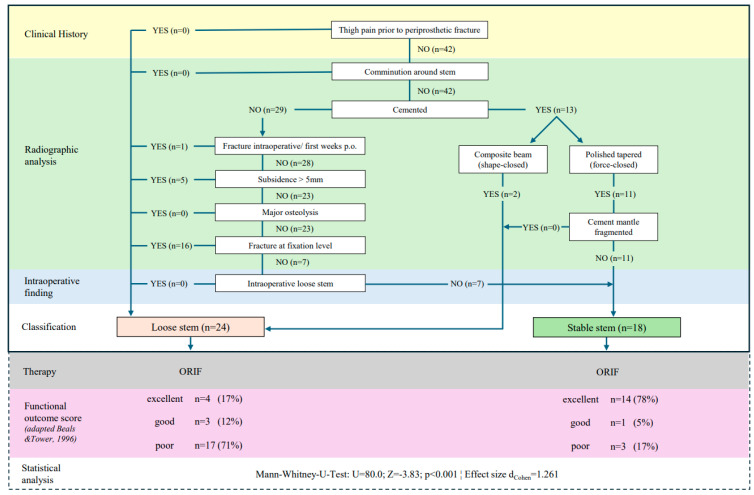
Beyond the Vancouver Classification [11] with the number of subjects throughout the algorithm and functional outcome measure [23].

As illustrated in Figure 2, an overview of the Beyond the Vancouver Classification is provided, showing the number of subjects in the population at each stage of the algorithm and the resulting functional outcome score between B2 ‘loose’ stems and B2 ‘stable’ stems. Of the 42 Vancouver B2 fractures that were initially classified, 13 were with cemented stems and 29 with cementless stems. Intraoperative examination revealed that none of the 29 cementless stems exhibited signs of loosening. The re-classification of patients according to the Beyond Vancouver classification resulted in the identification of 24 B2 ‘loose’ stems, while 18 were categorized as B2 ‘stable’.

Statistical analysis revealed significant differences in the functional outcome score (adapted by Beals and Tower) between the B2 ‘loose’ and B2 ‘stable’ group (U = 80.0, Z = −3.83, *p* < 0.001), with a large effect size of d_Cohen_ =1.261. The results for B2 ‘loose’ stems treated with ORIF were found to be poor in 71% (n = 17) of patients, but excellent or good in 29% (n = 7) of patients. The outcomes for B2 ‘stable’ stems treated with ORIF were either excellent or good in 83% (n = 15) of patients, while 17% (n = 3) of patients exhibited poor outcomes.

The descriptive analysis of the secondary outcome parameters is shown in Table 3, subdivided for the total sample and for the two subgroups B2 ‘stable’ and B2 ‘loose’. In the present study, 53% of patients with PPFs treated with ORIF revealed an excellent or good functional outcome score (adapted by Beals and Tower). The complication rate was 22%, including infections and conversion to revision arthroplasty. The fractures healed in 95% of cases. The study found that 81% of patients reported minimal or no pain, 87% were able to ambulate independently or with the assistance of a cane or walker, and 26% were able to ambulate without restriction. Further details can be found in Table 3.

## 4. Discussion

The management of periprosthetic femur fractures is initially determined by the Unified Classification System (UCS) [5], which suggests that well-fixed stems (type B1) can be treated with a plate (ORIF), while loose stems (types B2 and B3) should be treated with a revision [25]. Determining whether a stem is loose is not always straightforward, which has prompted us to extend the existing UCS with an adapted algorithm that has been validated in this retrospective analysis. In summary, the results validated the proposed algorithm and thus the hypotheses: ORIF was associated with favorable outcomes in 83% of patients with stems re-classified as stable. Notably, 29% of patients with stems re-classified as loose demonstrated excellent or good results.

### 4.1. B2 ‘Stable’ Stems

Recently, the use of the UCS to cemented polished tapered stems, which, by definition, are loose within their cement mantle has been the subject of recent discourse [26,27,28]. Lower reliability and validity of the UCS when applied to polished stems have been reported [26]. Maggs et al. [29] proposed a modification of the UCS classification for polished stems, with the additional aspect of whether the cement-bone interface is well fixed or loose. However, they still recommend revision arthroplasty for all B2 fractures. Conversely, other authors have concluded that favorable outcomes in B2 fractures with cemented polished stems can be attained with ORIF, provided the reduction in the bone and the cement mantle are perfectly anatomical. However, it has been demonstrated that in certain fracture patterns, such as comminuted metaphyseal split fractures, it is not feasible to achieve anatomical reduction and stable fixation. Consequently, these fractures should be treated with revision arthroplasty [28,30]. The aforementioned aspects have been incorporated into the Beyond the Vancouver Classification, and their suitability is reflected in the results, which indicate that seven of nine patients with polished tapered stems treated with ORIF achieved excellent or good clinical outcomes. In two cases, the patients experienced adverse outcomes as a result of infection and non-union.

The influence of the fracture pattern on the treatment strategy has also been reported for uncemented stems. As demonstrated previously, fractures occurring outside the region of the initial fixation of the stem [31] can be categorized as stable and consequently treated with ORIF [4,32]. Karam et al. (2020) added the awareness of distinct fracture patterns, which will prove useful to surgeons in predicting stem stability [33]. The incorporation of this assumption into the Beyond the Vancouver Classification resulted in the identification of seven cases as stable, with six of these cases resulting in excellent or good outcomes following ORIF. One patient exhibited unsatisfactory results due to an implant-associated infection and implant loosening, necessitating subsequent revision.

### 4.2. B2 ‘Loose’ Stems

**Figure 3:**
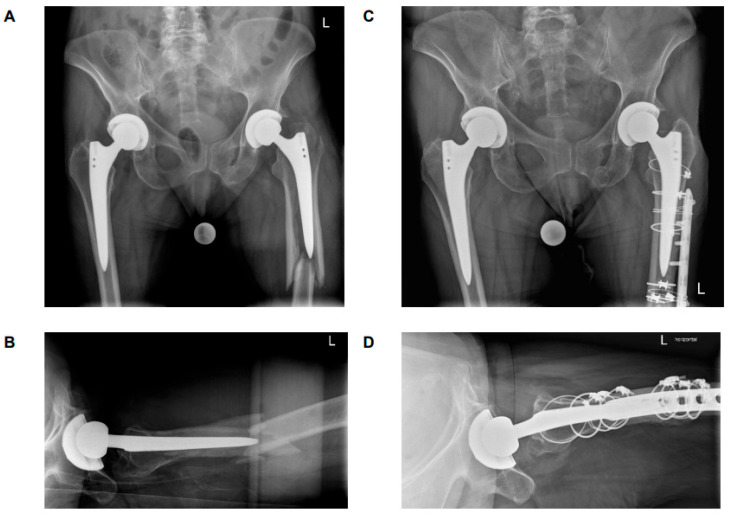
Antero-posterior and axial X-rays. (**A**,**B**) preoperatively; (**C**,**D**) 52-week-follow-up. A 71-year-old women with PPF, left, ORIF, excellent functional outcome. By definition, the stem is loose, but as the greater trochanter is still attached to the prosthesis, the patient has no limb and no stem subsidence.

**Figure 4:**
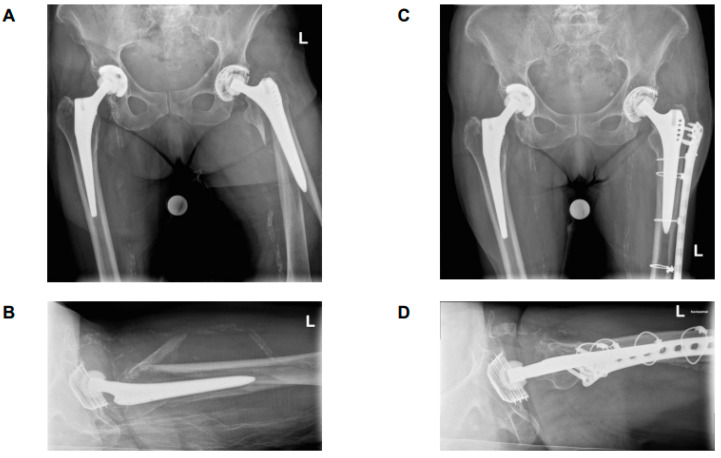
Antero-posterior and axial X-rays. (**A**,**B**) preoperatively; (**C**,**D**) 18-week-follow-up. A 93-year-old men with PPF, left, ORIF, excellent functional outcome. By definition, the stem is loose, but as the greater trochanter is still attached to the prosthesis, the patient has no limb and no stem subsidence.

It is noteworthy that among the patients classified as B2 ‘loose’, who were anticipated to result in a poor outcome, 29% of cases were found to have an excellent (n = 4) or good (n = 3) outcome. A thorough investigation of these patients revealed a consistent fracture pattern, characterized by an intact large fracture fragment of the greater trochanter and the lateral cortical bone connected to the stem along its entire length. As demonstrated in Figure 3 and Figure 4, two patients with this fracture pattern who achieved an excellent outcome following treatment with ORIF. It has been hypothesized that this particular fracture pattern is responsible for the favorable outcome observed, both in terms of pain management and functional capacity. One potential explanation for this result is that the fragment can be anatomically reduced and optimally surgically fixed. A further salient reason for this result could be that the insertions of the main hip abductors are located on this particular fragment, thereby enabling them to retain their function due to the integrity of the fragment. It can be hypothesized that this may reduce the likelihood of leg shortening due to stem subsidence. However, it may also provide a rationale for the favorable functional outcome observed in patients. As indicated by previous studies, hip muscle strength has been demonstrated to exert a significant influence on functional outcome. It is therefore recommended that surgeons give due consideration to this factor when determining surgical procedures [34]. Postoperative reduction in hip abductor strength due to damage to the muscular structures can affect abductor function and the patient’s ability to walk normally [35]. It can thus be concluded that the preservation of muscular structures is of crucial importance in terms of functional outcome and rehabilitation [36]. It was observed that all patients who achieved an excellent or good result, despite being categorized as B2 ‘loose’, had a stem with distal fixation.

**Figure 5:**
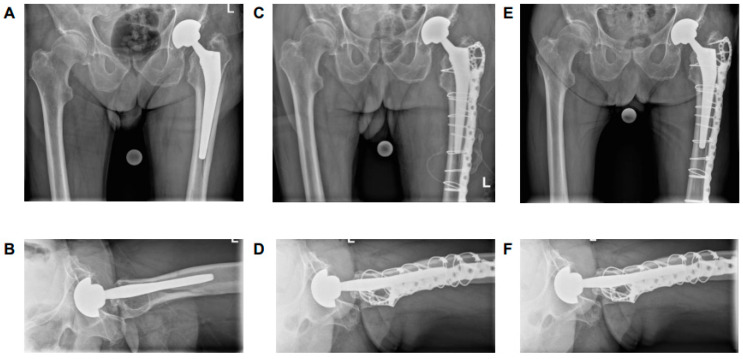
Antero-posterior and axial X-rays. (**A**,**B**) preoperatively; C-D postoperatively; E-F 12-week-follow-up. A 89-year-old men with PPF, left, sintered stem (18 mm) preoperatively (**A**,**B**), nearly anatomical reduction and fixation of the PPF during surgery (**C**,**D**), 12-week FU sintered (17 mm) to preoperative level (**E**,**F**) with a poor outcome including weakness and thigh pain.

In contrast, all patients with a poor outcome confirm the algorithm according to which a fracture at the fixation level of the stem or a sintered stem should be categorized as ‘loose’ and therefore treated with a revision arthroplasty. As demonstrated in Figure 5, the radiological images of a patient for whom no favorable outcome could be achieved with ORIF treatment are presented. Despite the successful reduction in the stem, which had already been sintered prior to surgery, it had returned to its previous level by the 18-week follow-up. In this context, however, it must be recognized that other factors not yet included in the algorithm can also lead to a poor outcome. For instance, patient age, reduced bone quality, comorbidities, or an excessively invasive surgical approach may also be associated with an unfavorable outcome.

**Figure 6:**
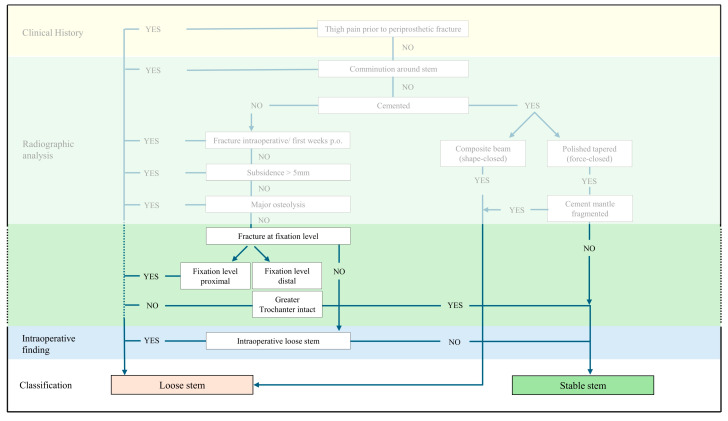
Beyond the Vancouver Classification [11] modification based on the fixation level and fracture pattern in cementless stems.

The results of this study indicate that it would be beneficial to supplement the existing Beyond the Vancouver Classification with the described specific fracture pattern: Distally fixed stems with an intact greater trochanter and a lateral cortical bone attached to the stem can be classified as B2 ‘stable’ and treated with ORIF, even if the fracture is at the level of stem fixation. The proposed modification to the Beyond the Vancouver Classification is illustrated in Figure 6.

### 4.3. Strength and Limitations

This study demonstrates the efficacy of plate treatment for Vancouver B2 fractures, thereby contributing to the validation of the modified UCS classification proposed by Stoffel et al. (2020) [11]. It is proposed that the algorithm be further specified in relation to the fracture pattern, as indicated by the findings of this case series. This specification is expected to facilitate the decision-making process for the appropriate treatment regime. The generalizability of the results of the study is limited due to the retrospective design of the study, which was conducted at a single center. The number of patients and the relatively brief follow-up period for some of them may be considered a limitation of the study. This issue may be addressed by larger studies in the future. The exclusive inclusion of patients who received an ORIF for their fracture may result in heterogeneity within the study population and an unequal distribution between cemented and cementless stems. This may have resulted in the falsification of the results. The primary objective of this cohort study was not primarily to ascertain statistical group differences, but rather to analyze for which Vancouver type B2 fractures ORIF may be the treatment of choice.

## 5. Conclusions

It is suggested by this case series that certain Vancouver B2 fractures can be treated with ORIF and achieve excellent or good outcomes. The Beyond the Vancouver classification system may provide a framework for the categorization of ‘stable’ or ‘loose’ stems. The validity of the algorithm was supported by the observation that ORIF yielded excellent and good results for the majority of ‘stable’ classified stems, but poor results for the majority of stems classified as ‘loose’. Moreover, it has been demonstrated that the fracture pattern is a crucial factor that should be considered when treating distally fixed cementless stems. The classification was consequently broadened to encompass the particular fracture patterns exhibited in cementless distally fixed stems, which can be effectively treated with ORIF. In view of the findings of this study, it is recommended that polished tapered stems with an intact cement mantle be classified as Vancouver Type B2 ‘stable’. It is suggested that the Beyond the Vancouver classification may provide further guidance in the identification of ‘loose’ or ‘stable’ stems.

It is crucial to acknowledge that achieving optimal outcomes with ORIF in cemented polished stems is contingent on the precise anatomical reduction in the bone within an intact cement mantle. Furthermore, it is imperative to acknowledge that the post-operative management protocol differs depending on the surgical approach employed, namely ORIF or revision surgery. The immobility and non-loading that characterize these interventions can have a significant impact on patient morbidity and mortality. In selecting an appropriate therapeutic approach, it is critical to consider the patient’s morbidity as a secondary criterion. In order to ensure anatomical reduction and avoid infection, it is imperative that the algorithm criteria are consistently applied and that the procedure is performed by a surgeon who is appropriately qualified.

## Figures and Tables

**Table 1:** Patient characteristics.

Age at fracture (years)	Median (range) 83 (73–88) years
Gender	Number (%)
Female	25 (60%)
Male	17 (40%)
ASA classification	ASA 1: 1 (2%)
	ASA 2: 19 (45%)
	ASA 3: 22 (52%)
Type of primary stem	Number (%)
Cemented	12 (29%)
Cementless	30 (71%)
Lifetime of existing prosthesis	Median (range) 78 months (4–134 months)
Time between ORIF and last follow-up	Median (range) 24 weeks (12–36 weeks)

**Table 2:** Description of the functional outcome measure (adapted with permission from S. Tower, Clinical Orthopaedics and Related Research, 1996) [23].

	Clinical				Functional		
	Arthroplasty		Fracture and Complication		Mobility		Pain
Excellent	Stable (no Osteolysis, Subsidence <5 mm)	and	Healed Minimal deformity No shortening	and	Limp-free Unrestricted walking or short distances out of home	and	No or minimal pain
Good	Stable Subsidence (5–10 mm)	or	Healed Moderate deformity Moderate shortening	or	Slight limp Rollator Short distances at home	or	Little pain
Poor	Loose Subsidence > 10 mm	or	Non-union Sepsis New Fracture Severe deformity Severe shortening	or	Limping Wheelchair	or	Pain

**Table 3:** Overall, and stratified (B2 ‘loose’, B2 ‘stable’) radiographic and clinical results.

Parameter	Number (%)	Number (%)	Number (%)
	Overall (n = 42)	B2 ‘loose’ (n = 24)	B2 ‘stable’ (n = 18)
Overall Functional Outcome Score *			
Excellent	18 (43%)	4 (17%)	14 (78%)
Good	4 (10%)	3 (13%)	1 (6%)
Poor	20 (48%)	17 (71%)	3 (17%)
Complications postoperative			
Infection	4 (10%)	2 (8%)	2 (11%)
Revision	5 (12%)	4 (17%)	1 (6%)
Stem subsidence at time of fracture			
>5 mm	4 (10%)	4 (17%)	0 (0%)
Stem subsidence at follow-up			
<5 mm	21 (50%)	6 (25%)	15 (83%)
5–10 mm	6 (14%)	3 (13%)	3 (17%)
>10 mm	15 (36%)	15 (63%)	0 (0%)
Osteolysis	2 (5%)	2 (8%)	0 (0%)
Fracture healed	40 (95%)	24 (100%)	16 (89%)
Pain			
No pain	26 (62%)	11 (46%)	15 (83%)
Little pain	8 (19%)	6 (25%)	2 (11%)
Pain	8 (19%)	7 (29%)	1 (6%)
Mobility Walking aids - Without aids - One cane - Two canes - Walker - Wheelchair Walking distance - Unrestricted walking - Short distances outside of home - Short distances at home - Not able to walk			
		
	16 (38%)	7 (29%)	9 (50%)
	6 (14%)	4 (17%)	2 (11%)
	6 (14%)	3 (13%)	3 (17%)
	9 (21%)	5 (21%)	4 (22%)
	5 (12%)	5 (21%)	0 (0%)
		
		
	11 (26%)	4 (17%)	7 (39%)
	24 (57%)	14 (58%)	10 (56%)
	2 (5%)	1 (4%)	1 (6%)
	5 (12%)	5 (21%)	0 (0%)
		

* adapted from Beals and Tower [23].

## Data Availability

The data presented in this study are available on request from the corresponding author. The data are not publicly available due to ethical restrictions.
